# Sparse Reconstruction of Sound Field Using Bayesian Compressive Sensing and Equivalent Source Method

**DOI:** 10.3390/s23125666

**Published:** 2023-06-17

**Authors:** Yue Xiao, Lei Yuan, Junyu Wang, Wenxin Hu, Ruimin Sun

**Affiliations:** School of Mechanical Engineering, Nanchang Institute of Technology, Nanchang 330099, China; yuanlei1027@163.com (L.Y.); wangjunyu65@163.com (J.W.); 13617056713@163.com (W.H.); 18379672851@163.com (R.S.)

**Keywords:** near-field acoustic holography, Bayesian compressive sensing, equivalent source method

## Abstract

To solve the problem of sound field reconstruction with fewer measurement points, a sound field reconstruction method based on Bayesian compressive sensing is proposed. In this method, a sound field reconstruction model based on a combination of the equivalent source method and sparse Bayesian compressive sensing is established. The MacKay iteration of the relevant vector machine is used to infer the hyperparameters and estimate the maximum a posteriori probability of both the sound source strength and noise variance. The optimal solution for sparse coefficients with an equivalent sound source is determined to achieve the sparse reconstruction of the sound field. The numerical simulation results demonstrate that the proposed method has higher accuracy over the entire frequency range compared to the equivalent source method, indicating a better reconstruction performance and wider frequency applicability with undersampling. Moreover, in environments with low signal-to-noise ratios, the proposed method exhibits significantly lower reconstruction errors than the equivalent source method, indicating a superior anti-noise performance and greater robustness in sound field reconstruction. The experimental results further verify the superiority and reliability of the proposed method for sound field reconstruction with limited measurement points.

## 1. Introduction

Near-field acoustic holography (NAH) is a robust technology that was first introduced in the early 1980s and has been proven to be an effective tool for identifying sound sources and reconstructing sound fields [1,2,3]. According to different principles of sound field spatial transformation, various NAH algorithms have been developed, such as the spatial Fourier transform method [4], the boundary element method [5], and the equivalent source method (ESM) [6]. Among them, the ESM can be applied to sound sources of any shape and has significant advantages in terms of calculation accuracy and efficiency. This method uses a superimposed sound field generated by several equivalent sound sources to replace the radiated sound field of the actual sound sources. The source strengths of the equivalent sources can be determined by matching the acoustic pressures measured on the hologram surface [7,8].

The solution of NAH is to treat the acoustic radiation problem as an inverse problem. By measuring the sound pressure data on the hologram surface, it is possible to directly reconstruct the sound pressure and normal velocity of the sound source surface. This information can then be used to predict the radiation characteristics of the entire three-dimensional sound field. An inverse problem is considered ill-posed if its solution fails to meet the criteria of existence, uniqueness, and stability. Currently, regularization techniques are commonly employed to mitigate the influence of measurement errors and stabilize the solution process for inverse sound field reconstruction problems. The Tikhonov regularization method [9] is a commonly used technique for obtaining stable solutions to inverse problems. However, its reconstruction effectiveness is primarily limited to low and medium frequencies and its performance can be significantly reduced at high frequencies. Fortunately, the sound field reconstruction technology based on the Bayesian regularization algorithm can provide stable solutions at low and medium frequencies, and also demonstrates a good reconstruction performance at high frequencies.

However, all of the aforementioned conventional NAH algorithms require a large number of measurement points to be arranged on the holographic surface in order to satisfy the Nyquist sampling theorem, thus achieving relatively accurate sound source localization and sound field reconstruction results. Increases in experimental costs hinder the practical application and promotion of this technique in engineering. Recently, the compressive sensing (CS) theory [10] has revealed that signals that were originally sparse or sparse in certain transform domains can be represented by only a few nonzero coefficients, and the original signal can be well reconstructed from a limited number of measurement points using sparse reconstruction algorithms. The CS theory reduces the number of required sampling sensors, thereby decreasing the experimental costs of the acoustic source identification system, which effectively addresses the limitations of the Nyquist sampling theorem and provides a novel approach for implementing the NAH technique [11]. The field of CS theory primarily involves three essential components: the sparse representation of a signal, the creation of a measurement matrix that meets the requirement for randomness, and the development of a reconstruction algorithm that can effectively and accurately recover the original signal.

The key to CS technology lies in the design of reconstruction algorithms. In recent years, sparse regularization methods have gained attention as important tools for enhancing resolution in various fields, especially in image processing. Sparse regularization methods have also been applied in the field of sound source reconstruction. Currently, there are several algorithms that are used for CS reconstruction, including greedy iterative algorithms [12,13,14], convex optimization algorithms [15,16,17], iterative hard thresholding (IHT) algorithms [18,19], and sparse Bayesian algorithms. Greedy algorithms do not prioritize global optimization, instead focusing on optimizing the objective function incrementally through iterations. Greedy iterative algorithms tend to produce locally optimal solutions. The convex optimization algorithm employs the restricted isometry property (RIP) [20] to transform the difficult task of minimizing the *l*_0_ norm into a more computationally feasible problem, that is, minimizing the *l*_1_ norm by using the equivalence between the measurement matrix of the *l*_0_ norm and *l*_1_ norm. The algorithm has the capability to achieve a globally optimal solution with high accuracy, but its complexity results in slow operation speed. Additionally, the measurement matrix must strictly adhere to the RIP constraint, which can make it unsuitable for many practical engineering applications. The IHT algorithm is not a convex optimization method and is similar to the orthogonal matching pursuit (OMP) algorithm [21]. This algorithm is faster than the convex optimization algorithm for signal recovery and its computational complexity is equivalent to that of the OMP algorithm. In the sparse Bayesian algorithm, the unknown parameter vector that needs to be estimated is treated as a random vector that conforms to a prior distribution. The prior distribution is determined based on prior knowledge of the parameters. According to the given sample data, the posterior probability distribution can be calculated using the Bayesian rule. The unknown parameters can be deduced by integrating prior information and posterior probabilities. Inferring unknown parameters is a fundamental problem in algorithm design. With the development of sparse Bayesian theory, it has gained attention and found applications in an increasing number of fields. Some scholars use a support vector machine (SVM) [22] to select a kernel function for inferring unknown parameters. However, the kernel function used in the SVM must satisfy Mercer’s condition. Despite their effectiveness, some challenges must be faced when training SVM models, such as high computational requirements and low sparsity. In 2001, Tipping proposed a sparse probabilistic model, the relevance vector machine (RVM) [23], to address the deficiencies of SVMs. In 2008, Ji et al. [24] proposed a Bayesian compressive sensing (BCS) framework for estimating the underlying signal based on compressive measurements. Babacan et al. [25] used Laplace prior distribution to sparsify signals and implemented greedy algorithms for the fast reconstruction of the sound field, thereby improving the reconstruction accuracy through Bayesian compressive sensing. Ning et al. [26] enhanced the spatial resolution of sound sources using a robust Bayesian super-resolution method that enforces strong sparsity priors. Huang et al. [27] developed a robust Bayesian compressive sensing algorithm for approximately sparse signals by combining the features from monitoring data. Pereira et al. [28] employed Bayesian methods to address the difficulty of automatically selecting appropriate regularization parameters to solve acoustic inverse problems. Bush et al. [29] proposed a phenomenological model based on Bayesian inference for broadband coprime microphone array responses to broadband noise sources, which achieved accurate estimation of the direction of arrival of the sound source and prediction of the number of sound sources. Niu et al. [30] proposed a block sparse Bayesian learning method using the dispersion relation to estimate multi-frequency horizontal wavenumbers. In their work, they analyzed the horizontal wavenumbers within a specific frequency range using data from a vertical line array and sound source localization was achieved without prior knowledge of the bottom parameters or source information.

The purpose of this paper is to achieve the high-accuracy reconstruction (i.e., prediction) of sound fields while significantly reducing the number of required measurement points (undersampling). Conventional NAH is based on the Nyquist sampling theorem, and its reconstruction performance depends on the number of sampling points. Achieving high-accuracy sound field prediction requires expensive measurement costs. In the present work, a robust sound field reconstruction model based on a combination of the equivalent source method and sparse Bayesian compressive sensing is proposed to overcome the limitations of the Nyquist sampling theorem. By utilizing a relevant vector machine to estimate the maximum a posteriori probability of the source strength and noise variance, an optimal solution for a sparse coefficient vector with equivalent source strength can be obtained. Once the equivalent source strength has been determined, the prediction of the sound field can be completed. In this method, the performance of signal recovery is improved because the proposed Bayesian compressive sensing algorithm fully takes into account the measurement noise in the model when reconstructing the sound field. Additionally, incorporating a hierarchical prior model in an RVM to improve sparsity and adjusting the function form of the prior distribution to align with the conjugate prior can result in a highly efficient Bayesian inference process. Furthermore, since all the required parameters in the BCS model are automatically estimated during the calculation process, the proposed algorithm is fully automated. Compared with conventional NAH based on the ESM using Tikhonov regularization [31], the proposed method can achieve higher accuracy of sound field reconstruction with undersampling.

The outline of this paper is as follows: Firstly, the application of NAH technology and CS in the field of sound source identification is briefly introduced. Section 2 introduces the basic theories of ESM and BCS. In Section 3, numerical simulations are performed to evaluate the proposed method by comparing it with conventional ESM. The effectiveness of the proposed method is further validated through experiments in Section 4. Finally, the conclusions are summarized in Section 5.

## 2. Theoretical Background

### 2.1. Equivalent Source Method-Based NAH

**Figure 1:**
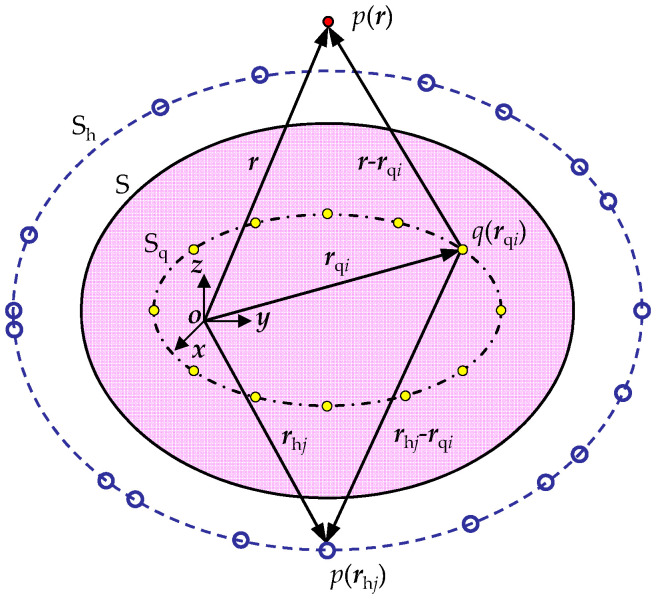
Schematic diagram of NAH based on ESM.

The sound pressure and particle velocity at any point in space can be approximated by several equivalent sources placed inside the sound source [6]. As shown in Figure 1, the hologram measurement surface S_h_ is placed near the outside of the sound source surface S, and the equivalent source surface S_q_ is located on the other side of the sound source surface S. This means that the sound pressure and particle velocity at point ***r*** in space are, respectively, expressed as

(1) $$p(r)=\sum_{i=1}^{N}i\rho ckg(r,r_{qi})q(r_{qi})$$

(2) $$v(r)=\sum_{i=1}^{N}-\frac{\partial g(r,r_{qi})}{\partial r}q(r_{qi})$$

where p(r) and v(r) are the sound pressure and particle velocity at point ***r***, while $q(r_{qi})$ and $r_{qi}$ are the source strength and position of the *i*-th equivalent source. k=ω/c is the wave number, ω is the angular frequency, *c* is the sound velocity, *N* is the total number of equivalent sources, and $g(r,r_{qi})=\exp(ik\left|r-r_{qi}\right|)/(4\pi \left|r-r_{qi}\right|)$ represents Green’s function in free space.

The sound pressures of *M* measurement points on the hologram measurement surface S_h_ can be expressed in matrix form as

(3) $$P_{h}=G_{\mathrm{sh}}q$$

where $P_{h}={\left[p(r_{\mathrm{h1}}),p(r_{\mathrm{h2}}),\cdots ,p(r_{hj}),\cdots ,p(r_{hM})\right]}^{T}$ is the pressure column vector at *M* measurement points on the hologram measurement surface S_h_, $r_{hj}$ is the location vector of the *j*-th microphone (j=1,2,⋯,M), $G_{\mathrm{sh}}(j,i)=i\rho ckg(r_{hj},r_{qi})$ is the transfer matrix between the equivalent source strength on the surface S_q_ and the measured sound pressure on the hologram measurement surface S_h_, and $g(r_{hj},r_{qi})=\exp(ik\left|r_{hj}-r_{qi}\right|)/(4\pi \left|r_{hj}-r_{qi}\right|)$, $q={\left[q(r_{\mathrm{q1}}),q(r_{\mathrm{q2}}),\cdots ,q(r_{qi}),\cdots ,q(r_{qN})\right]}^{T}$ is the equivalent source strength column vector.

According to Equation (3), the equivalent source strength can be calculated as

(4) $$q=G_{\mathrm{sh}}^{+}P_{h}$$

where “+” represents the generalized inverse of the transfer matrix.

The solution for the equivalent source strength ***q*** is an acoustic inverse problem. During the process of measuring sound pressure on the hologram measurement surface, some amount of measurement noise is inevitably introduced. Meanwhile, the transfer matrix ***G***_sh_ in Equation (3) is usually an ill-condition matrix. Directly inverting it can lead to excessive amplification of noise, which may result in an inaccurate solution.

The Tikhonov regularization method is commonly used to deal with such ill-posed problems, which is one of the most effective methods to obtain a more stable solution. The principle is to introduce the *l*_2_ norm of the solution into the objective function and obtain accurate results by balancing the residual norm of the solution and the norm of the solution as

(5) $$arg\;\underset{q}{min}\left({\left\|P_{h}-G_{\mathrm{sh}}q\right\|}_{2}^{2}+\lambda {\left\|q\right\|}_{2}^{2}\right)$$

where λ is the regularization parameter, which can be obtained via the generalized cross validation (GCV) method.

Furthermore, in order to ensure that unique solutions are obtained, conventional NAH generally requires that the number of measurement points *M* is larger than the number of equivalent sources *N*, i.e., the problem is overdetermined.

The sound pressure and particle velocity on any reconstruction surface in the sound field can be determined by obtaining the equivalent source strength as

(6) $$P_{r}=G_{sr}q$$

(7) $$V_{r}=G_{vr}q$$

where $G_{sr}$ and $G_{vr}$ represent the sound pressure transfer matrix and the particle velocity transfer matrix from the sound source surface to the reconstructed surface, respectively.

### 2.2. Compressive Sensing Theory

The conventional NAH method is based on the Nyquist sampling theorem and has limitations on its reconstruction accuracy due to the spatial sampling rate (i.e., the distance between microphones). Therefore, conventional NAH usually requires a large number of sensors to achieve a good reconstruction performance. Fortunately, the compressive sensing theory overcomes the limitations of the Nyquist sampling law in signal reconstruction by using the sparsity of signals in a certain transform domain and provides an innovative idea for reconstructing sound fields using undersampling. It can achieve high-accuracy signal reconstruction with a much lower sampling rate compared to the Nyquist sampling method and can significantly reduce the number of required sensors and measurement data, leading to a reduction in measurement costs and workload [10].

Let the original signal $x\in R^{N}$ on an orthonormal basis $\Psi =[\psi_{1},\psi_{2},\cdots ,\psi_{N}]$ be represented as

(8) x=Ψs+ε

where the vector $s\in R^{N}$ has only *K* (*K*≪*N*) non-zero values, ε is the noise, and ***s*** and Ψ are the sparse coefficient vector and sparse basis matrix of signal ***x***, respectively.

In compressive sensing theory, the measurement vector ***y*** is observed by linearly measuring the original signal using a measurement matrix. The essence of this involves completing the compressed mapping from high-dimensional signals to low-dimensional space via linear measurements since the number of measurement points *M* is much smaller than the signal dimension *N*. A stable and reliable measurement matrix ensures that the measurement value ***y*** contains all the information from the original signal ***x*** without losing any data due to the reduction in dimensions. Thus, a measurement matrix $\Phi \in R^{M\times N}$ (*M*≪*N*) independent of the sparse basis can be used to perform compression observations of the original signal ***x***, that is, the measured signal ***y*** is represented as

(9) y=Φx+ε

where $y\in R^{M}$ is the measurement vector.

Substituting Equation (10) into Equation (11) can be obtained by

(10) y=ΦΨs+ε=Θs+ε

where Θ is an M×N matrix.

When ***x*** is compressible, the solution problem of Equation (10) is transformed into an optimization problem of the minimum *l*_0_ norm. However, *l*_0_ norm optimization is numerically difficult to calculate, so *l*_1_ norm optimization is used as an alternative:

(11) $$arg\;\underset{s}{min}\left({\left\|y-\Theta s\right\|}_{2}^{2}+\lambda {\left\|s\right\|}_{1}\right)$$

### 2.3. ESM Based on the CS Theory

The transfer matrix $G_{sr}$ in Equation (6) can be decomposed by singular value decomposition (SVD); then, Equation (6) can be rewritten as

(12) $$P_{r}=USV^{H}q$$

where ***U*** and ***V*** are the left and right singular vectors of the transfer matrix $G_{sr}$, respectively, and both are mutually orthogonal unitary matrices; S is a diagonal matrix composed of positive singular values; and the upper corner script “H” represents the conjugate transpose.

Take the right-singular vector of the transfer matrix as the basis matrix of sparse reconstruction, i.e., Ψ=V [16]. Then, the equivalent source strength under the compressive sensing framework can be sparsely represented as

(13) q=Vw

where ***w*** is the sparse coefficient vector with equivalent source strength.

By substituting Equation (13) into Equation (3), the hologram measurement signal can be expressed as

(14) $$P_{h}=G_{sh}Vw+\varepsilon =Hw+\varepsilon$$

where $H=G_{sh}V$ is the sensing matrix.

When the number of measurement points *M* is smaller than the number of equivalent sources *N*, the problem of solving the sound source becomes an underdetermined problem and an accurate reconstructed signal cannot be obtained. Through CS theory, the problem of minimizing the *l*_2_ norm in Equation (5) can be transformed into the problem of minimizing the *l*_1_ norm [17]. The sparse coefficient vector with equivalent source strength w can be equivalent to the following *l*_1_ norm minimization problem as

(15) $$arg\;\underset{w}{min}\left({\left\|P_{h}-Hw\right\|}_{2}^{2}+\lambda {\left\|w\right\|}_{1}\right)$$

### 2.4. Bayesian Model via RVM

The Bayesian learning process involves using prior probabilities of parameters and posterior probabilities derived from sample information to obtain a full probability density function. By assuming prior probabilities, the overall distribution can be inferred. The specific expressions of prior probability and sample information in Bayesian learning theory are joint probability and conditional probability, respectively. Bayesian theory is a fundamental approach to statistical modelling, relying on the Bayesian formula to combine priori information, population and sample to derive the posterior distribution of unknown parameters. The process can be summarized as follows: First, the prior information and its distribution are established, along with the corresponding conditional probability density function. Secondly, the Bayesian formula is used to convert the prior distribution into the posterior probability density function. Finally, a relevant statistical decision or inference can be made based on the posterior probability.

In order to improve the computational efficiency of the Bayesian model, an RVM was employed to estimate the maximum posterior probability of the original signal in this paper. Recently, RVMs have been applied to text recognition [32], image classification [33], timing analysis [34], and other fields. In an RVM, a type-II maximum-likelihood (ML) procedure is considered with the objective of achieving highly efficient computations while still maintaining accurate results. In addition, according to Equation (14), the prior density function of the signal with respect to parameters ***w*** and ***ε*** can be constructed. The construction of the prior density function can be divided into the Laplace prior and Gaussian prior. However, because the Laplace prior is not conjugate to the Gaussian likelihood, the Bayesian inference cannot be iterated in closed form. In this paper, a hierarchical prior model of the RVM is introduced, and the prior distribution of ***w*** is assumed to follow a Gaussian distribution. In this way, the RVM not only has the prior property of the Laplace, but it can also perform conjugate exponential analysis.

**Figure 2:**
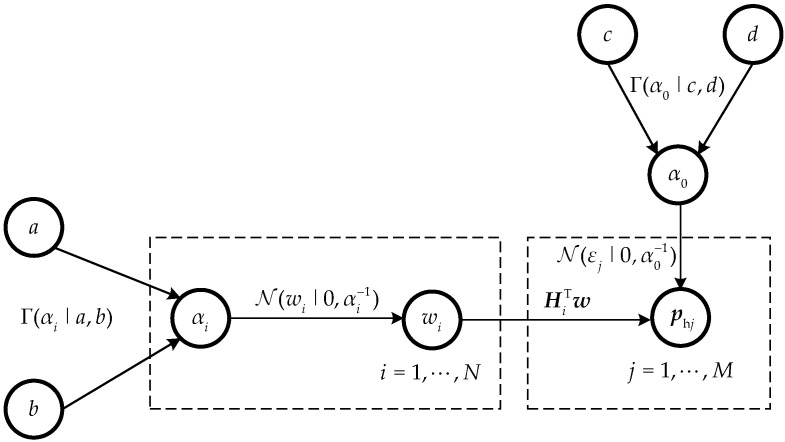
Graphical model of Bayesian compressive sensing.

In the RVM framework, if the prior distributions and hyperparameters are known, the mean and variance expressions can be obtained. Then, the marginal distribution of the hyperparameters is maximized, and the update expression for the hyperparameters is obtained using the MacKay iteration method. After repeated operations, specific conditions are reached and then stopped. Figure 2 shows the process of iterative implementation for the evaluation of the posterior density function of ***w*** and *α*_0_, where *α_i_* and *α*_0_ represent the hyperparameters of the prior distribution of the sparse coefficient vector with equivalent source strength ***w*** and measurement error ***ε***, respectively, which can be obtained through the Gamma prior distribution. $\Gamma (\alpha_{i}|a,b)$ is the Gamma distribution, where *a* is the shape parameter and *b* is the scale parameter of *α_i_*. $\Gamma (\alpha_{0}|c,d)$ is the Gamma distribution, where *c* is the shape parameter and *d* is the scale parameter of *α*_0_. $N(w_{i}|0,\alpha_{i}^{-1})$ and $N(\varepsilon_{i}|0,\alpha_{0}^{-1})$ are the zero-mean Gaussian distributions and their variances are $\alpha_{i}^{-1}$ and $\alpha_{0}^{-1}$, respectively.

When there is a lack of prior knowledge about the distribution of a certain parameter and uncertainty about which form to choose, Gaussian distribution is a suitable default option for two reasons. Firstly, most distributions, in reality, are actually rather close to a Gaussian distribution. The central limit theorem demonstrates that the sum of numerous independent random variables approximates a Gaussian distribution. Among all probability distributions with the same variance, the entropy of Gaussian distribution is the largest. When the data distribution is unknown, the model with the maximum entropy is usually chosen. Therefore, Gaussian distribution can be regarded as the distribution that contributes minimum prior knowledge to the model. Additionally, the mathematical properties of Gaussian distribution are excellent. Gaussian distribution has continuity, differentiability, and monotonicity in its probability density function. It exhibits the property of maximum likelihood estimation, which allows for the estimation of its parameters using this method. In a sparse Bayesian model, the prior distribution for measurement error ***ε*** and a sparse coefficient vector with equivalent source strength ***w*** are determined based on the principle of maximum likelihood estimation. Considering its broad applicability and exceptional mathematical properties, it is reasonable to use Gaussian distribution as the probability distribution for both the measurement error and the sparse coefficient vector with equivalent source strength in this paper.

Note that choosing Gamma distribution as the prior distribution for the hyperparameters *α_i_* and *α*_0_ is that Gamma distribution is the conjugate prior distribution of Gaussian distribution, which can cause the posterior distribution to have the same functional form as the prior distribution, but with different parameters. Since the prior distribution of measurement error ***ε*** and the sparse coefficient vector with equivalent source strength ***w*** were approximated as Gaussian distributions, it is possible to avoid the large number of calculations required to update the posterior distribution, making the calculation process highly efficient and convenient. In fact, assuming that the prior distribution of the hyperparameters *α_i_* and *α*_0_ follows an uninformative prior, which implies that all values are equally likely, is equivalent to assuming a uniform distribution. However, in the work of Tipping et al. [23], it was proven that assigning certain deterministic prior distributions to hyperparameters *α_i_* and *α*_0_ can promote sparse solutions. Gamma distribution was introduced due to the convenience of probability derivation. It belongs to the same exponential distribution family as Gaussian distribution and can maintain the same form in the derivation process, depending only on the key parameters.

A zero-mean Gaussian prior [23] on each element of ***w*** can be defined as

(16) $$P\left(w|\alpha \right)=\prod_{i=1}^{N}N\left(w_{i}|0,\alpha_{i}^{-1}\right)$$

where ***α*** represents the hyperparameter of the prior distribution of the sparse coefficient vector with equivalent source strength.

Further, a Gamma prior is considered over ***α***

(17) $$P\left(\alpha |a,b\right)=\prod_{i=1}^{N}\Gamma \left(\alpha_{i}|a,b\right)$$

By marginalizing over hyperparameter ***α***, the overall prior on ***w*** is then evaluated as

(18) $$P\left(w|a,b\right)=\prod_{i=1}^{N}\int_{0}^{\infty }N\left(w_{i}|0,\alpha_{i}^{-1}\right)\Gamma \left(\alpha_{i}|a,b\right)d\alpha_{i}$$

The density function $\Gamma (\alpha_{i}|a,b)$ is the conjugate prior for *α_i_*, while *w_i_* plays the role of observed data and $N(w_{i}|0,\alpha_{i}^{-1})$ is a likelihood function.

### 2.5. Sound Field Reconstruction Based on Bayesian Compressive Sensing

In traditional Bayesian compressive sensing theory, the sparse solution of Equation (14) can be obtained directly through the sparse Bayesian learning algorithm. However, the sparse Bayesian learning algorithm is based on a probability distribution solution for signal processing, which is not applicable to complex signals, and cannot independently calculate the real and imaginary parts of holographic complex sound pressure. Therefore, the solutions for both the real and imaginary parts of each element in Equation (14) can be rewritten as

(19) P=GQ+d

with $P=\left[\begin{array}{c} Re\left(P_{h}\right)\\ Im\left(P_{h}\right) \end{array}\right]$, G=ReH−ImHImHReH, Q=RewImw, d=ReεImε, $P\in R^{2M}$, $G\in R^{2M\times 2N}$, $Q\in R^{2N}$, $d\in R^{2M}$

where Re(·) and Im(·) denote taking the real and imaginary parts of the corresponding quantities, respectively. Both the real and imaginary parts of the measurement error can be considered as following a zero-mean Gaussian distribution with homoscedasticity.

According to the central limit theorem, assuming that both the real and imaginary parts of the measurement error satisfy a zero-mean Gaussian distribution with the precision (inverse-variance) of $\beta^{2}=1/\alpha_{0}$, the likelihood function of ***P*** can be written as

(20) $$P\left(P|Q,\beta^{2}\right)={\left(2\pi \beta^{2}\right)}^{-M}\exp\left(-\frac{1}{2\beta^{2}}{\left(P-GQ\right)}^{T}(P-GQ)\right)$$

where $\beta^{2}$ represents the hyperparameter of the prior distribution of the measurement error.

The sparse Bayesian learning algorithm controls the sparsity of the sparse coefficient vector with equivalent source strength ***Q*** by setting its hyperparameter ***α*** and each element ***Q****_i_* approximates as a zero-mean Gaussian prior distribution with the variance $\alpha_{i}^{-1}$

(21) $$P\left(Q|\alpha \right)=\prod_{i=1}^{N}N\left(Q_{i}|0,\alpha_{i}^{-1}\right)={\left(2\pi \right)}^{-N}{\left|A\right|}^{\frac{1}{2}}\exp\left(-\frac{1}{2}Q^{T}AQ\right)$$

where $A=\mathrm{diag}(\alpha_{1},\alpha_{2},\cdots \alpha_{N},\alpha_{1},\alpha_{2},\cdots \alpha_{N})$, $\alpha =\left[\alpha_{1},\alpha_{2},\cdots \alpha_{N}\right]$ determines the prior distribution of the sparse coefficient vector with equivalent source strength ***Q***.

When ***α*** and *β*^2^ are unknown, solving the sparse coefficient vector with equivalent source strength ***Q*** can transform into a problem of maximizing the joint posterior probability $P\left(Q,\alpha ,\beta^{2}|P\right)$ by combining Equations (20) and (21).

Decompose the joint posterior probability $P\left(Q,\alpha ,\beta^{2}|P\right)$ into

(22) $$P\left(Q,\alpha ,\beta^{2}|P\right)=P\left(Q|P,\alpha ,\beta^{2}\right)P\left(\alpha ,\beta^{2}|P\right)$$

where the posterior probability distribution $P\left(Q|P,\alpha ,\beta^{2}\right)$ of ***Q*** can be expressed by the Bayesian theorem as

(23) $$P\left(Q|P,\alpha ,\beta^{2}\right)=\frac{P\left(P|Q,\beta^{2}\right)P\left(Q|\alpha \right)}{P\left(P|\alpha ,\beta^{2}\right)}$$

where the denominator $P\left(P|\alpha ,\beta^{2}\right)$ can obtained by performing marginal integration on the sparse coefficient vector with equivalent source strength ***Q*** using the likelihood function

(24) $$P\left(P|\alpha ,\beta^{2}\right)=\int P\left(P|Q,\beta^{2}\right)P\left(Q|\alpha \right)dQ={\left(2\pi \right)}^{-M}{\left|C\right|}^{-\frac{1}{2}}\exp\left(-\frac{1}{2}P^{T}{\left(C\right)}^{-1}P\right)$$

with $C=\beta^{2}Ι+GA^{-1}G^{T}$.

Substituting Equations (20), (21) and (24) into Equation (23) yields

(25) $$P\left(Q|P,\alpha ,\beta^{2}\right)={\left(2\pi \right)}^{-N}{\left|\Sigma \right|}^{-\frac{1}{2}}\exp\left(-\frac{1}{2}{\left(Q-\mu \right)}^{T}\Sigma^{-1}(Q-\mu )\right)$$

where the posterior covariance and mean of the Gaussian distribution of ***Q*** [27] are, respectively,

(26) $$\Sigma ={\left(\beta^{-2}G^{T}G+A\right)}^{-1}$$

(27) $$\mu =\beta^{-2}\Sigma G^{T}P$$

According to the Bayesian theorem [26], if a known measurement data point ***P*** is given, the corresponding posterior probability density function can be expressed as:

(28) $$\begin{array}{ccc} P\left(\alpha ,\beta^{2}|P\right) & = & \frac{P\left(P|\alpha ,\beta^{2}\right)P\left(\alpha ,\beta^{2}\right)}{P\left(P\right)}\\ & \propto & P\left(P|\alpha ,\beta^{2}\right)P\left(\alpha ,\beta^{2}\right)\\ & \propto & P\left(P|\alpha ,\beta^{2}\right)P\left(\alpha \right)P\left(\beta^{2}\right) \end{array}$$

where “∝“ stands for the “proportional” sign, while $P\left(P\right)$ is the normalization constant, which is usually not taken into consideration in the solving process to simplify the calculation without affecting the parameter optimization performance. The process of Bayesian inference focuses on the distribution of parameters within the interval range, rather than on the specific parameter values.

Based on Equation (28), ***α*** and *β*^2^ can be predicted by maximizing $P\left(P|\alpha ,\beta^{2}\right)$. The maximization problem $P\left(P|\alpha ,\beta^{2}\right)$ in Equation (24) can be converted into logarithmic form

(29) $$\log\left(P\left(P|\alpha ,\beta^{2}\right)\right)=-\frac{1}{2}\left[2M\log\left(2\pi \right)+\log\left|C\right|+P^{T}{\left(C\right)}^{-1}P\right]$$

Equation (29) is used to calculate the partial derivative of ***α*** and *β*^2^, respectively, and make the derivative equal to zero. The updated formula can be obtained according to the MacKay method [23] as

(30) $$\alpha_{i}^{\mathrm{new}}=\frac{\gamma_{i}}{\mu_{i}^{2}},i\in \left\{1,2,\cdots ,N\right\}$$

where $\gamma_{i}=1-{\left[\Sigma \right]}_{i,i}\alpha_{i}$, ${\left[\Sigma \right]}_{i,i}$ is the *i*-th diagonal element of ***Σ***.

(31) $${\left(\beta^{2}\right)}^{\mathrm{new}}=\frac{{\left\|P-G\Sigma \right\|}_{2}^{2}}{2N-\sum_{i=1}^{N}\gamma_{i}}$$

**Figure 3:**
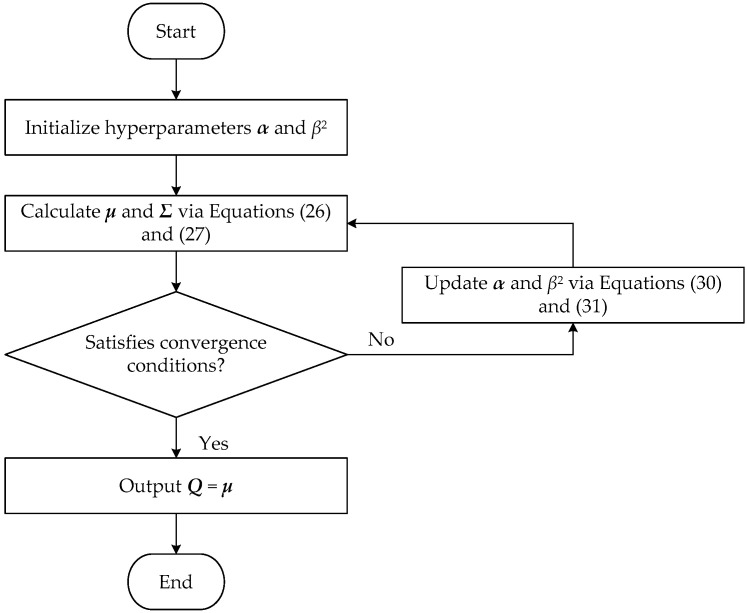
Flow diagram of sparse Bayesian learning.

The initial values of ***α*** and *β*^2^ are set, respectively, and are substituted into Equations (26) and (27) to update ***Σ*** and ***μ***. Then, ***α*** and *β*^2^ are re-estimated with updated ***Σ*** and ***μ*** values according to Equations (30) and (31). The above calculation is repeated until ***α*** and *β*^2^ satisfy certain convergence conditions to complete the estimation of ***Σ*** and ***μ***. In the actual calculation process, most of *α_i_* tends to be infinite, and the corresponding sparse coefficient vector with equivalent source strength ***Q****_i_* approaches zero, thus realizing the sparse model. The algorithm flow chart is shown in Figure 3.

The sparse vector ***w*** can be expressed as

(32) $$w=\mu \left(1:N\right)+i\mu \left(N+1:2N\right)$$

where $i=\sqrt{-1}$ is the imaginary unit.

This paper mainly focuses on the problem of solving the sparse coefficient vector with equivalent source strength in a complex model. The specific steps of the BCS model proposed in this paper for the sparse reconstruction of the sound field are summarized as follows:

1. Take a measurement of complex sound pressure on the hologram measurement surface and then convert the complex model into a real model using Equation (19), which can be processed using Bayesian theory;
2. Approximate the measured sound pressure of the real model as a Gaussian likelihood distribution using Equation (20);
3. Initialize the values of hyperparameters ***α*** and *β*^2^ in the Bayesian learning process;
4. Calculate ***Σ*** and ***μ*** using Equations (26) and (27), where ***Σ*** and ***μ*** are the covariance and mean of the posterior distribution of the sparse coefficient vector ***Q***, respectively;
5. Use the MacKay algorithm to iteratively calculate the updated hyperparameters using Equations (30) and (31);
6. Determine whether the iteration result satisfies the iteration stopping condition. If it does not, repeat iteration update steps 4 and 5 until the condition is satisfied and the iteration stops;
7. Obtain the final estimation of the sparse coefficient vector with equivalent source strength ***Q*** = ***μ***;
8. Convert the sparse coefficient vector ***Q*** from the real model into the sparse coefficient vector with equivalent source strength ***w*** in the complex model using Equation (32);
9. Calculate the equivalent source strength ***q*** using Equation (13);
10. Determine the sound pressure and particle velocity of the reconstruction surface using Equations (6) and (7), thereby allowing sound field reconstruction to be achieved.

## 3. Numerical Simulations

**Figure 4:**
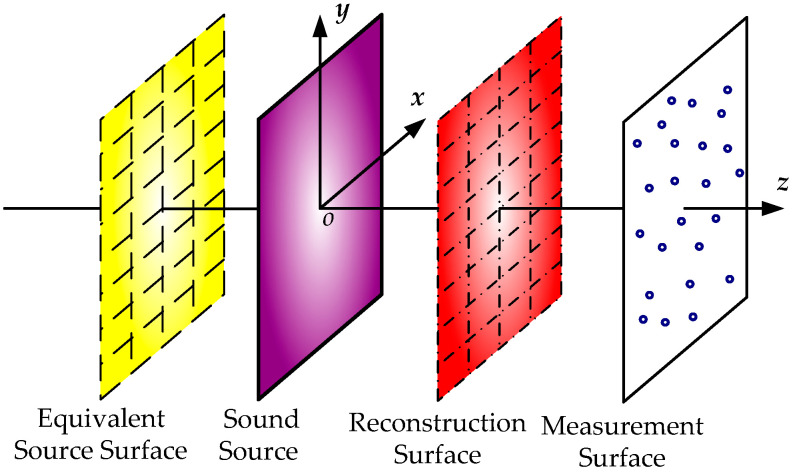
The layout of the simulations for source reconstruction.

**Figure 5:**
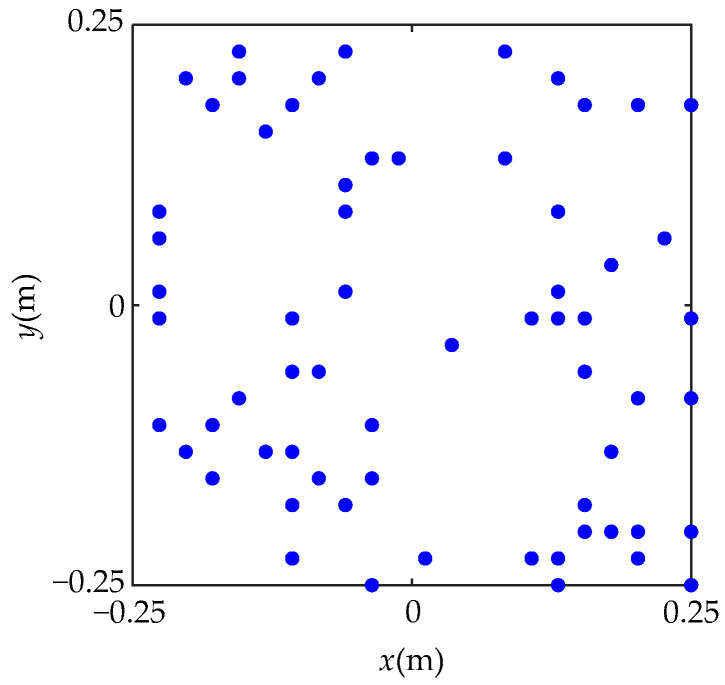
The simulated microphone array.

Numerical simulations were implemented to evaluate the performance of the proposed method. A simple, supported steel plate with a thickness of 3 mm and a size of 50 × 50 cm^2^ was used as the sound source in the simulation. A harmonic force with an amplitude of 1 N was applied to excite the steel plate at its central position. The origin of the coordinates was set at the central position of the plate, as shown in Figure 4. Poisson’s ratio of the steel plate was 0.28, the density of steel plate was 7.85 × 10^3^ kg/m^3^, and Young’s modulus was 2.1 × 10^11^ Pa. The measurement surface was placed parallel to the steel plate at a distance of 5 cm, with a sampling interval of 2.5 cm and a size of 50 × 50 cm^2^. Simulated pressures were measured via a square random planar array containing 64 microphones, as shown in Figure 5. Gaussian white noise with a signal-to-noise ratio (SNR) of 30 dB was added to the measured pressures. The reconstruction surface was the same size as the measurement surface and located 2 cm away from the plate. The equivalent source surface was set to be 5 cm away from the surface of the plate and the size of the equivalent source surface was equal to that of the above surfaces. In the simulation, hyperparameters ***α****_i_* and *β*^2^ are initialized as $\alpha_{i}^{\mathrm{initial}}=10^{8}$ and ${\left(\beta^{2}\right)}^{\mathrm{initial}}=0.1\times \mathrm{var}(P)$, respectively, where var(·) denotes the variance. The convergence criterion is considered to be satisfied during the updating process when $\mathrm{lg}\left(\alpha_{i}^{\mathrm{new}}\right)-\mathrm{lg}\left(\alpha_{i}\right)<10^{-4}$ and $\mathrm{lg}\left({\left(\beta^{2}\right)}^{\mathrm{new}}\right)-\mathrm{lg}\left(\beta^{2}\right)<10^{-6}$.

To quantify the reconstruction accuracy of the sound pressure on the reconstruction surface, the relative error of reconstruction is defined as

(33) $$\eta =\frac{{\left\|P_{r}-P_{\mathrm{th}}\right\|}_{2}}{{\left\|P_{\mathrm{th}}\right\|}_{2}}\times 100\%$$

where $P_{r}$ and $P_{\mathrm{th}}$ are the reconstructed and theoretical sound pressure (or particle velocity), respectively.

**Figure 6:**
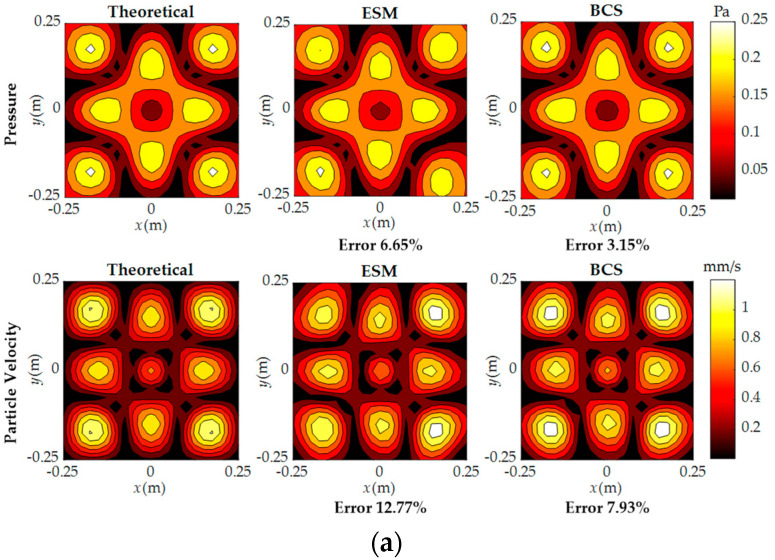

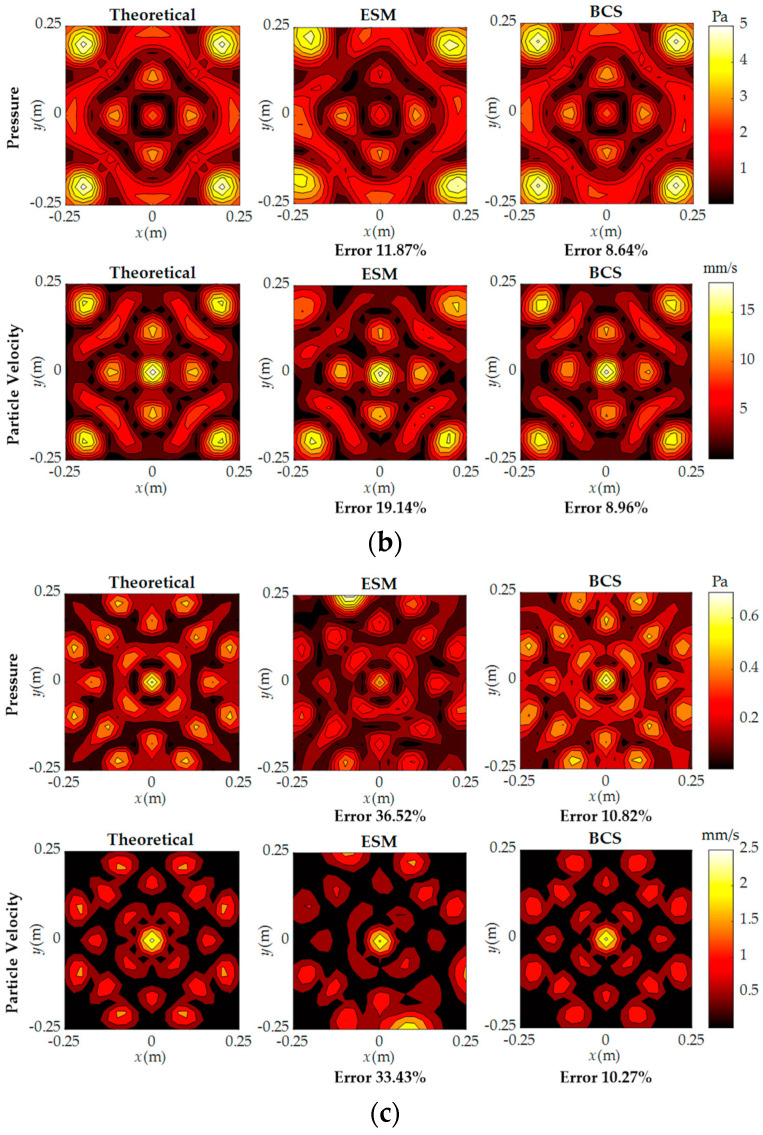
Comparison of reconstructed pressure and particle velocity with 64 sampling points at three frequencies: (**a**) 500 Hz; (**b**) 1000 Hz; (**c**) 1500 Hz.

The reconstruction was performed at 500 Hz, 1000 Hz, and 1500 Hz using 64 samplings, which were randomly selected from 441 samplings on the measurement surface. The theoretical and reconstructed pressure and particle velocity are compared in Figure 6. As shown in Figure 6a, when the frequency is 500 Hz, both the ESM and BCS methods have very small reconstruction errors. However, the reconstruction accuracy of the BCS method is higher than that of the ESM method at low frequencies. It can be seen from Figure 6b that when the frequency is 1000 Hz, the peak distribution and symmetrical distribution characteristics on the reconstruction surface by the ESM method are not reconstructed, while the BCS method accurately reconstructs the peak distribution and symmetrical distribution characteristics, reflecting that the reconstruction effect of BCS is still better than that of the ESM method at medium frequencies. In Figure 6c, when the frequency is increased to 1500 Hz, the ESM method cannot accurately reconstruct the distribution of sound pressure and particle velocity due to the insufficient number of measurement points. In contrast, the BCS method can reconstruct the overall details and peak positions, demonstrating high consistency with the theoretical values. The reconstruction performance of BCS is still much better than that of the ESM at high frequencies. Overall, it can be seen that good reconstruction results can be obtained by BCS, whether at low or high frequencies, and all reconstruction errors by BCS can be maintained at around 10%, indicating that the BCS method can effectively achieve sound field reconstruction with fewer measurement points in the whole frequency range.

**Figure 7:**
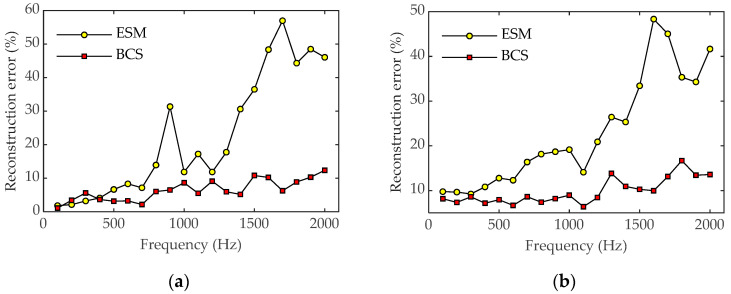
Reconstruction errors of the two methods versus frequency with 64 sampling points. (**a**) Pressure; (**b**) particle velocity.

Figure 7 shows the reconstruction errors of sound pressure and particle velocity using the ESM and BCS, respectively, over a frequency range between 100 and 2000 Hz. As can be seen from Figure 7, the relative errors of sound pressure and particle velocity reconstructed by BCS fluctuate steadily throughout the frequency range. The error value of the reconstructed sound pressure is mostly kept below 10%, and the error value of the reconstructed particle velocity is around 10–20%. In contrast, the reconstruction errors of sound pressure and particle velocity using the ESM method show an overall increasing trend with increasing frequency. By comparing the errors at each frequency, it can be seen that the error of BCS is lower than that of the ESM. Under the condition of reducing the number of measurement points, BCS can not only effectively reconstruct sound field information, but also improve the reconstruction accuracy compared with the sparse reconstruction of the sound field by the ESM.

**Figure 8:**
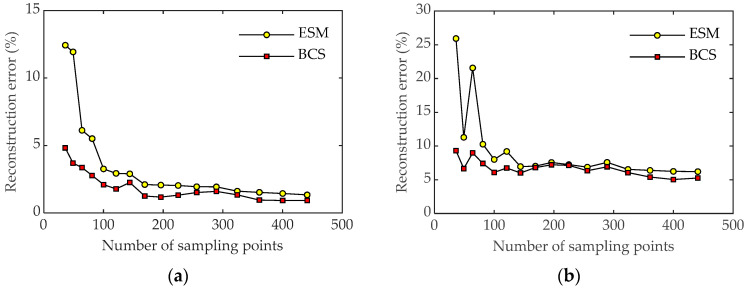

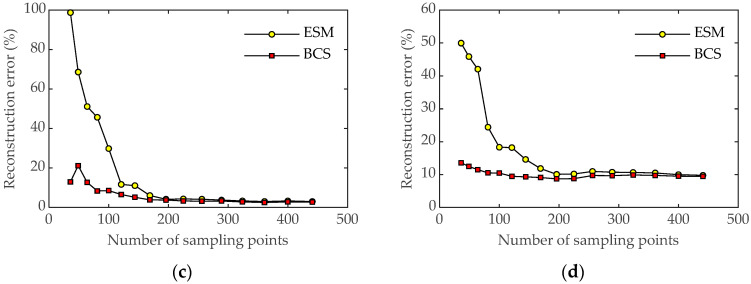
Reconstruction errors of the two methods with an SNR of 30 dB under different numbers of sampling points. (**a**) Pressure at 600 Hz; (**b**) particle velocity at 600 Hz; (**c**) pressure at 1600 Hz; (**d**) particle velocity at 1600 Hz.

The influence of the number of sampling points on the reconstructed results was also investigated. Figure 8 shows the reconstruction errors of the two methods with the variation in the number of sampling points at 600 Hz and 1600 Hz. As can be seen from Figure 8, when the number of measurement points increases, the reconstruction errors for both methods change slightly, indicating that increasing the number of samplings can effectively improve the reconstruction accuracy of the sound field. At a low frequency, the reconstruction accuracy of the two methods is relatively low, and the error is less than 10%. However, at a high frequency, the error distribution obtained by the ESM method is not as good as that obtained by the BCS method when there are few measurement points. When the number of measurement points is small, the error results obtained by the ESM method are mostly above 30%, while the reconstruction error obtained by the BCS method remains at about 10%. It can be concluded that the BCS method has good reconstruction accuracy at both low and high frequencies with sparse sampling points.

**Figure 9:**
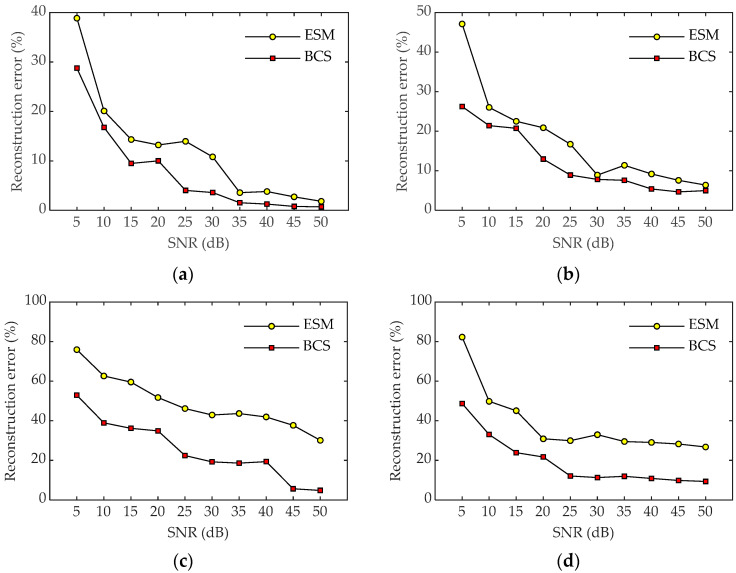
Reconstruction errors of the two methods under different SNRs with 64 sampling points. (**a**) Pressure at 600 Hz; (**b**) particle velocity at 600 Hz; (**c**) pressure at 1600 Hz; (**d**) particle velocity at 1600 Hz.

Furthermore, the effect of the SNR on the reconstruction results is also studied. Figure 9 shows the reconstruction errors of sound pressure and particle velocity for the two methods at 600 Hz and 1600 Hz with 64 measurement points under different SNRs. As can be seen from Figure 9, the reconstruction errors of the two methods gradually decrease with the increase in the SNR, and the error of BCS is generally smaller than that of the ESM. Although the reconstruction error of the ESM is not much different from that of BCS at 600 Hz, the reconstruction error of BCS is obviously better than those of the ESM at 1600 Hz, indicating that BCS has better anti-noise performance and is more robust in sound field reconstruction.

## 4. Experiment

**Figure 10:**
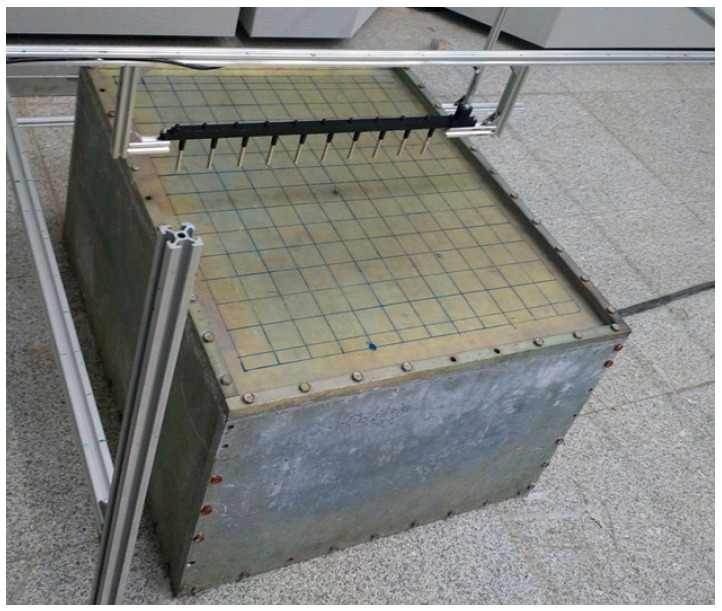
The experimental setup.

An experiment was carried out in a semi-anechoic chamber to further examine the performance of the proposed method. The experimental setup is shown in Figure 10. The experiment used a vibrating steel plate with dimensions of 0.65 × 0.8 m^2^ and a thickness of 3 mm as the sound source. The plate was excited by a harmonic force applied by a vibrator, and the excitation signal was composed of a series of sine signals with a frequency range from 200 to 1000 Hz and a frequency interval of 100 Hz. The hologram surface and reconstruction surface were located 0.1 m and 0.05 m away from the steel plate, respectively, with the same dimensions of 0.6 × 0.75 m^2^ and a uniform spatial interval of 0.05 m. The pressures were measured on the hologram surface using a linear array with a grid of 13 × 16 and a total of 208 sampling points. The pressures at 208 points on the reconstruction surface were reconstructed using 81 sampling points, which were randomly selected from the pressures at 208 original points on the hologram surface. The pressures at these 208 points on the reconstruction surface were also measured to serve as the theoretical pressures for comparing the reconstruction performance of various methods. The equivalent source surface was arranged 0.05 m behind the surface of the steel plate.

**Figure 11:**
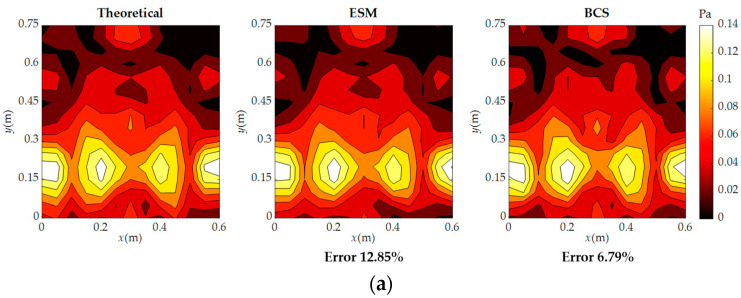

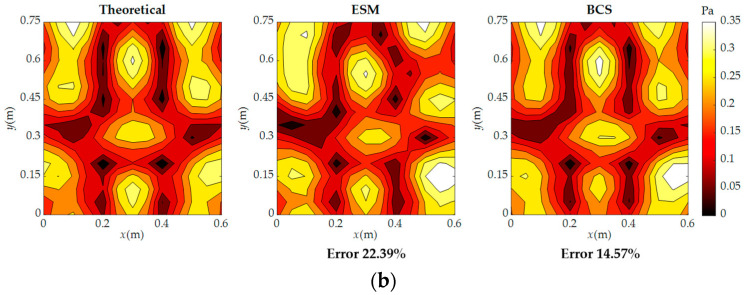
Comparison of reconstructed and theoretical pressure with 81 sampling points at two frequencies: (**a**) 500 Hz; (**b**) 900 Hz.

Figure 11 shows a comparison of the reconstructed and theoretical pressure at 500 Hz and 900 Hz. It can be seen that when the frequency is 500 Hz, both the ESM and BCS can achieve good agreement with the theoretical values, with a reconstruction error of 12.85% for the ESM and 6.79% for BCS. However, when the frequency is 900 Hz, the ESM with 81 sampling points is not sufficient for accurate reconstruction, resulting in a reconstruction error of 22.39%. On the other hand, BCS has a reconstruction error of only 14.57%, and the reconstructed sound pressure distribution still has high consistency with the theoretical values, indicating that the proposed method has a better sound field reconstruction performance than the ESM when the number of measurement points is small.

**Figure 12:**
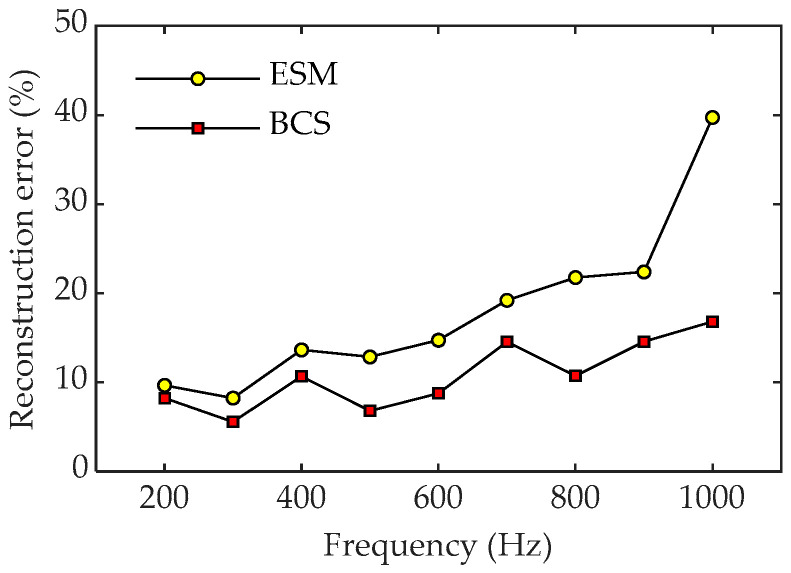
Reconstruction errors of the two methods versus frequency with 81 sampling points.

Figure 12 shows a comparison of the pressure reconstruction errors for the two methods over a frequency range between 200 and 1000 Hz. It can be seen that when using 81 sampling points, the ESM obtains acceptable results at low frequencies, while it has larger reconstruction errors at high frequencies, meaning that more sampling points may be required to achieve greater reconstruction accuracy. In contrast, all reconstruction errors using BCS are mostly below 15%, especially the reconstruction error at low frequencies—which is less than 10%—indicating a better reconstruction performance by BCS across the whole frequency range.

**Figure 13:**
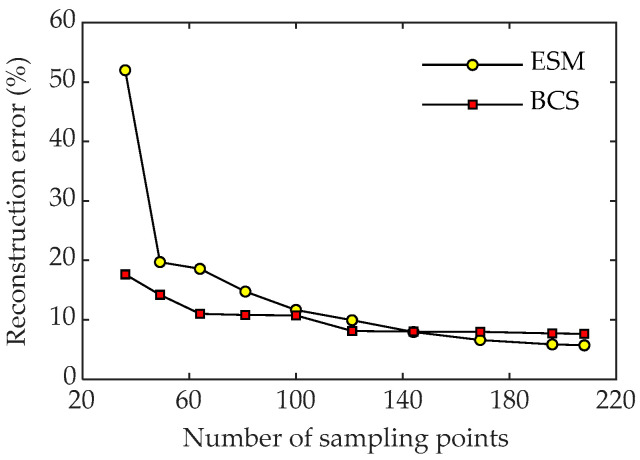
Reconstruction errors of the two methods with different numbers of sampling points at 700 Hz.

Figure 13 shows the pressure reconstruction errors of the two methods at 700 Hz using different numbers of sampling points. It can be seen that the reconstruction errors of the two methods gradually decrease with the increase in the number of measurement points. When there are fewer measurement points, the pressure error reconstructed by the ESM can even reach 50%. Under the same conditions, the reconstruction error of BCS is less than 20%. Furthermore, the reconstruction error of BCS is lower than that of the ESM and the change is very stable, indicating that BCS has better robustness than the ESM in reconstructing the sound field.

## 5. Conclusions

A sound field reconstruction method based on Bayesian compressive sensing is proposed in this paper. The sound field reconstruction of near-field acoustic holography based on the equivalent source method is transformed into a maximum a posteriori estimation problem of source strength and noise variance under the Bayesian framework. By combining the Bayesian principle with compressive sensing theory, the MacKay iteration of an RVM in the sparse Bayesian model was used to infer hyperparameters, the maximum a posteriori of the original signal was estimated, and the solution of the sparse coefficient with an equivalent source strength was obtained. The numerical simulation results demonstrate that the reconstruction error of the BCS method is about 10% across the whole frequency range, which is an improvement over the ESM, verifying that the proposed method has a wider range of frequency applicability. When the number of measurement points is limited, the BCS method exhibits a reconstruction error of approximately 10% at high frequencies and less than 5% at low frequencies, indicating that the proposed method can achieve higher accuracy compared to the ESM in undersampling conditions. The proposed method shows promise in significantly reducing measurement costs while maintaining high reconstruction accuracy in engineering applications. In addition, the BCS method demonstrates significantly higher reconstruction accuracy than the ESM in a low-SNR environment, indicating that the proposed method has a better anti-noise performance and greater robustness in sound field reconstruction. The experimental results also show that the BCS method outperforms the ESM in terms of its reconstruction performance. The reconstruction error of the BCS method is mostly below 15%, especially at low frequencies, where it is typically below 10%. Despite having fewer measurement points, the BCS method still achieves higher reconstruction accuracy than the ESM. This further verifies the stability and reliability of the proposed method in the sound field.

In future work, NAH based on a hierarchical block sparse Bayesian compressive sensing algorithm will be investigated to improve the efficiency and accuracy of sound field reconstruction. Moreover, we will explore the potential of utilizing Bayesian compressive sensing for active noise control. This will involve optimizing the update mechanism of the filter group, developing an improved cluster control method, and applying these techniques to reduce noise in various settings, such as in active soundproof windows and vehicle cabins, thereby expanding the scope of applications for Bayesian compressive sensing.

## Data Availability

Not applicable.
